# Patient-reported experience measures (PREMs), coercion, and mental health outcomes in psychiatric inpatient care: a national mixed-methods study comparing immigrant and majority populations

**DOI:** 10.1186/s12913-026-15511-0

**Published:** 2026-09-12

**Authors:** Lina Harvold Ellingsen-Dalskau, Øyvind Bjertnæs, Marte Karoline Råberg Kjøllesdal, Hilde Hestad Iversen

**Affiliations:** 1 Department of Health Services Research, Norwegian Institute of Public Health, PO Box 222, Oslo, Norway; 2 Department of Public Health Science, Norwegian University of Life Sciences, PO Box 5003, Ås, Norway

**Keywords:** Patient-reported experience measures, PREMs, Coercion, Mental health outcomes, Psychiatric inpatient care, Immigrant health, Health services research

## Abstract

**Background:**

Immigrants are at increased risk of mental health problems but underutilize specialist services and may face barriers to high-quality care. Evidence regarding their experiences in psychiatric inpatient settings remains limited. This study examined differences in patient experiences, coercion, and change in mental health between immigrant groups and the majority population and considered challenges in measuring and comparing patient-reported experiences across groups.

**Methods:**

A national survey was conducted across Norwegian psychiatric inpatient institutions from January 2020 to June 2022. The Psychiatric Inpatient Patient Experience Questionnaire – Continuous Electronic Measurement (PIPEQ-CEM) was used with demographic, coercion, and mental health indicators. Linear regression examined associations between region-of-birth and key outcomes. Additionally, 390 comments from immigrant patients and 125 from Norwegian-born patients were thematically analyzed.

**Results:**

Data from 8,184 patients (89.5% Norwegian-born) revealed few group differences in overall patient experience scores. However, some immigrant groups rated aspects—including initial welcome, privacy, and treatment benefit—lower than the majority population, in addition to higher levels of coercion and less improvement in mental health. Thematic analysis revealed immigrant-specific experiences related to expectations and prior knowledge of psychiatric care, belonging and trust, communication, and access to information.

**Conclusions:**

While overall patient experience scores were largely similar, some immigrant groups reported higher coercion and less favorable mental health trajectories. Qualitative findings highlighted contextual factors that may shape how patients experience and report care. These results underline the importance of culturally sensitive psychiatric care delivery and culturally appropriate measurement of patient experiences across diverse populations.

**Supplementary Information:**

The online version contains supplementary material available at https://doi.org/10.1186/s12913-026-15511-0.

## Background

Mental health disorders account for a substantial proportion of patient interactions with healthcare services [1] and represent a key public health challenge for immigrant populations in multiple countries [2, 3]. Some immigrant groups may be at higher risk of developing mental health problems [3–5], which has also been found in Norway [2, 6–8]. Despite these findings, the immigrant population underutilizes specialist mental healthcare services [9]. Barriers to accessing mental healthcare include language and communication challenges, limited health literacy, family influence, and stigma surrounding mental illness [10–12].

When entering the healthcare system, all patients should receive the same quality of care based on their individual needs. A systematic review on patient satisfaction with psychiatric inpatient care identified few studies and inconsistent findings regarding the relationship between ethnicity and satisfaction [13]. Methodological weaknesses, including sampling biases, also limited the generalizability of the findings [13]. Similarly, a scoping review about the experiences of immigrant and majority population patients in psychiatric care found a limited number of comparative studies and provided no firm conclusions [14].

Nevertheless, although survey-based quantitative studies do not provide strong evidence for poorer patient experiences in mental health care among immigrants compared to others, qualitative studies suggest that immigrant patients encounter barriers in psychiatric care. These include lack of cultural understanding and respect for specific needs, as well as challenges in communication due to cultural differences and language barriers [12, 14]. Professionals working in mental healthcare across 16 European countries reported similar challenges [15], and in Norway, healthcare professionals working with immigrants experiencing mental health and substance use disorders identified challenges with treatment engagement to be linked to language difficulties, lack of culturally adapted services, and socioeconomic factors [16].

Several mechanisms may account for differences in patient experiences across ethnic and immigrant backgrounds. These include variations in how individuals assess and report their experiences including response tendencies [17, 18], differences in expectations of healthcare [18, 19], and actual disparities in the quality of care provided [17, 19–21]. Comparing patient-reported experiences across diverse population groups therefore raises important questions about both potential differences in care and how measures are interpreted across culturally diverse patient groups.

Finally, research indicates that immigrants are at a higher risk of experiencing coercion and involuntary admissions to psychiatric care [22, 23]. The use of coercion in psychiatric care influences patient satisfaction [13, 24], and involuntary admission has been associated with limited prior contact with psychiatric services and a lack of outpatient treatment before hospitalization [22, 23]. Therefore, coercion is not solely a consequence of individual circumstances but also reflects systemic barriers to healthcare access and structural deficiencies within the healthcare system.

In Norway, immigrants represent a growing segment of the population, accounting for 17.5% in 2026 [25]. Achieving equity in healthcare requires a comprehensive understanding of both access to services and the quality of care provided across different population groups [26]. Service users provide a unique perspective on the care they receive, which can help identify deficiencies [27]. However, research comparing immigrant patients’ experiences with those of the majority population has yielded inconclusive results, while qualitative studies highlight specific barriers related to immigrant status. Together, this literature points to a need for studies that combine quantitative comparisons of patient-reported experiences with qualitative insight into how patients describe and interpret care in psychiatric inpatient settings.

This study examines differences in patient-reported experience measures, perceived coercion and self-reported mental health among psychiatric inpatients from six immigrant groups and Norwegian-born patients, using both survey data and patient comments. Identifying and understanding potential differences is important for improving equity and quality in mental health service delivery.

## Methods

### Study design

This was a national mixed-methods study using a convergent design. Quantitative survey data and qualitative patient comments from the same data collection were analysed separately and integrated at the interpretation stage using a narrative approach [28]. Reporting was guided by the Good Reporting of A Mixed Methods Study (GRAMMS) recommendations [29].

### Participants and data collection

All public and private inpatient mental healthcare institutions with a contract with the regional health authorities in Norway were included. Regional contact persons were responsible for compiling lists of institutions and coordinating the survey. Each participating unit assigned a project manager to disseminate information, distribute login credentials to patients, and report survey progress. The study population included adult inpatients (age ≥ 18 years) receiving specialized inpatient psychiatric care in Norway. The data were collected with continuous electronic measurement from January 1st, 2020, to June 30th, 2022. Patients completed the questionnaire near the time of discharge.

### Instrument

Patient experiences were measured using the Psychiatric Inpatient Patient Experience Questionnaire – Continuous Electronic Measurement (PIPEQ-CEM), which is a generic questionnaire developed to be relevant for all inpatients in psychiatric care. The questionnaire consists of 19 items about inpatient experiences grouped into three subscales: Structure and facilities, Patient-centred interactions, and Intermediate outcomes. The subscales have demonstrated satisfactory reliability, and validity [30]. An open text-field also allows respondents to provide additional comments about their inpatient care. Furthermore, the questionnaire includes questions about admission, mental health, coercion, and sociodemographic variables. Last, a question regarding region of birth was used to divide the sample into the seven groups: Norway, other Nordic countries, Western Europe/North America/Oceania, Eastern Europe, Africa, Asia, and South and Central America.

### Statistical analysis

Statistical analyses were conducted using IBM SPSS 26, with the significance level set at 0.05. Chi-square tests were used to examine associations between region of birth and categorical variables, including demographic characteristics, experiences of coercion, and perceived mental health. One-way ANOVA was used to investigate mean differences in patient experiences across region of birth.

Multiple linear regression analyses were conducted to examine associations between the main exposure variable region of birth (“Norway” as the reference category) and the dependent variables, patient experiences, coercion, and change in perceived mental health. Two models were applied: the first adjusted for age and sex, the second additionally adjusted for educational level, marital status, and number of previous admissions. Assumptions of multiple linear regression (e.g. multicollinearity, independence of observations and normality) were tested, with no violations observed. The five dependent variables and the adjustment variables are described below.

The three main indicators of patient experience: Structure and Facilities, Person-centred Interaction, and Intermediate Outcomes, were scored on a 5-point response scale, which was converted into scores of 0, 25, 50, 75, and 100. The coercion sum score was computed by combining the three items measuring coercion and involuntary admission in the questionnaire. The item “Is the treatment voluntary or do you feel coerced into receiving it?” was first dichotomized, with responses 1–2 coded as 0 (no coercion) and 3–5 as 1 (coercion). This item was then combined with the two items: “Were you admitted against your will?” (No = 0, Yes = 1) and “Have you experienced coercion during this stay” (No = 0, Yes = 1), resulting in a sum score ranging from 0 (no coercion) to 3 (highest degree of coercion). The mental health change score was calculated using the two items “Perceived mental health the week prior to admission” and “Perceived mental health at discharge”. The items were scored from 1 (Very poor) to 5 (Very good). A raw change score was computed by subtracting the pre-admission score from the discharge score. This raw score was then recoded into a five-point scale ranging from 1 (Very negative change) to 5 (Very positive change), with 3 indicating no change. Adjustment variables were age (“18–24 years” as reference), sex (“Male” as reference), educational level (“Primary education” as reference), marital status (“Married or living with a partner” as reference) and number of previous admissions (“0 previous admissions” as reference). All categorical variables with three or more categories were dummy coded before inclusion in the models.

### Patient comments

Responses to the open text-field were also analyzed. All the 390 comments made by patients with immigrant background were included (66 from Nordic countries, 73 from Western Europe/North America, 74 from Eastern Europe, 43 from Africa, 108 from Asia, and 26 from South and Central America). For Norway, 125 comments, randomly drawn from the 3,369 available comments, were included. This number was comparable to the number of comments from the different immigrant groups, and was considered sufficient as a study by Iversen et al. [31] showed that useful themes emerge from clusters of as little as 50 patient comments collected in a similar setting. A total of 14 comments were excluded due to missing data on country of birth.

### Analysis of comments

Two researchers (LED and HHI) conducted the analysis. In the first step, comments were independently coded by both researchers based on sentiment (positive, negative, mixed, or neutral), before coming together to reach a consensus. A total of 32 neutral comments unrelated to mental health services were excluded. In the second step, each researcher independently identified and coded the content of the comments before reaching a joint agreement and organizing the codes into main themes. In the third step, the main themes of comments made by patients with an immigrant background were compared to those from patients born in Norway. Comments that thematically differed were re-examined and coded for content (step two), resulting in three main themes specifically relevant across the six immigrant groups.

## Results

### Sample characteristics

A total of 8,248 patients from 226 psychiatric inpatient units responded. Of these, 8,184 answered the question regarding region of birth and were categorized into the seven groups: Norway 7,325 (89.5%), other Nordic countries 160 (2%) Western Europe/North America/Oceania 134 (2%), Eastern Europe 171 (2.1%), Africa 119 (1.5%), Asia 212 (2.6%), and South and Central America 63 (0.8%). Demographic details show that 59.7% of respondents were female, 44.6% were aged 25–44 years, 70.7% were not married/living with a partner, and 49% had upper secondary school (Table 1).

There were several significant differences between the Norwegian-born patients and immigrant groups regarding the sample characteristics. Eastern Europe, Africa, and Asia had a higher proportion of patients under 45 years of age. In contrast, immigrants from Nordic countries and Western Europe/North America had a greater share of patients between 45 and 66 years. These latter groups also reported higher levels of education compared to patients born in Norway (Table 1).

**Table 1:** Characteristics and background variables of inpatients in psychiatric care according to region of birth

*N* (%)	Norway(*N* = 7,325)	Nordic countrieswithout Norway (*N* = 160)	Western Europe/ North America/Oceania (*N*= 134)	Eastern Europe (*N* = 171)	Africa (*N* = 119)	Asia including Turkey (*N* = 212)	South and Central America (*N* = 63)
**Gender**							
Female	4,370 (60.0)	90 (57.3)	79 (59.8)	102 (60.4)	56 (47.9)	121 (57.3)	41 (67.2)
Male	2,916 (40.0)	67 (42.7)	53 (40.2)	67 (39.6)	61 (52.1)	90 (42.7)	20 (32.8)
**Age**							
18–24	1,363 (18.7)	15 (9.4)	13 (9.8)	30 (17.6)	23 (19.5)	33 (15.6)	10 (16.1)
25–44	3,215 (44.0)	61 (38.4)	42 (31.8)	95 (55.9)*	70 (59.3)*	121 (57.3)*	34 (54.8)
45–66	2,345 (32.1)	76 (47.8)*	73 (55.3)*	44 (25.9)	24 (20.3)	56 (26.5)	18 (29.0)
67+	376 (5.2)	7 (4.4)	4 (3.0)	1 (0.6)	1 (0.8)	1 (0.5)*	0 (0.0)
**Married or living with a partner**							
Yes	2,118 (29.0)	56 (35.2)	47 (35.1)	64 (37.6)	22 (18.6)	57 (27.1)	26 (41.9)
No	5,175 (71.0)	103 (64.8)	87 (64.9)	106 (62.4)	96 (81.4)	153 (72.9)	36 (58.1)
**Education**							
Primary school	1,351 (18.6)	15 (9.4)	8 (6.0)*	15 (8.8)*	27 (22.7)	39 (19.2)	7 (11.1)
Upper secondary school	3,607 (49.5)	79 (49.4)	38 (28.4)*	82 (48.2)	62 (52.1)	87 (42.9)	26 (41.3)
University or college	2,322 (31.9)	66 (41.3)	88 (65.7)*	73 (42.9)*	30 (25.2)	77 (37.9)	30 (47.6)
**Previous admissions**							
0	2,129 (29.2)	55 (34.6)	43 (32.1)	68 (39.8)	30 (25.2)	86 (40.8)*	24 (38.7)
1	1,247 (17.1)	30 (18.9)	24 (17.9)	35 (20.5)	30 (25.2)	33 (15.6)	9 (14.5)
2	724 (9.9)	18 (11.3)	15 (11.2)	18 (10.5)	19 (16.0)	22 (10.4)	9 (14.5)
3–5	1,116 (15.3)	25 (15.7)	18 (13.4)	27 (15.8)	15 (12.6)	26 (12.3)	5 (8.1)
>5	2,073 (28.4)	31 (19.5)	34 (25.4)	23 (13.5)*	25 (21.0)	44 (20.9)	15 (24.2)

^a^ Differences between Norwegian-born patients and immigrant groups were investigated using a Chi square test.

* Indicates a significant difference between Norwegian-born and the other region-of-birth groups at the p < 0.05 level

### Patient experiences and region of birth

For all groups, Structure and facilities was rated highest, followed by Person-centred interactions, while Intermediate outcomes received the lowest ratings. No significant differences were found for these indicators between Norway and the other region-of-birth groups (Table 2).

When considering single items of patient experiences, patients from Eastern Europe rated being prepared for the time after discharge and receiving information about treatment options significantly higher than Norwegian-born patients. Similarly, patients from Africa reported significantly higher scores for being prepared for the time after discharge and expressed greater confidence that life would be better after discharge. On the other hand, they rated their initial welcome and the level of privacy significantly lower. Lastly, patients from Asia provided significantly higher ratings for receiving information about treatment options, but significantly lower scores for their initial welcome and overall benefit of the treatment.

**Table 2:** Mean and standard deviation of indicators and single items of patient experiences according to region of birth

Indicators and items	Norway (*N* = 7,325)	Nordic countries without Norway (*N* = 160)	Western Europe North America Oceania (*N* = 134)	Eastern Europe (*N* = 171)	Africa (*N* = 119)	Asia including Turkey (*N* = 212)	South and Central America (*N* = 63)
**Structure and Facilities**	75.6 (17.0)	76.5 (16.9)	76.3 (16.5)	77.1 (19.5)	71.4 (20.2)	73.4 (19.9)	75.7 (17.3)
4. Satisfactory welcome to institution	81.0 (22.3)	82.5 (20.7)	80.1 (24.3)	81.1 (27.0)	68.1 (30.2)***	73.7 (25.1)***	80.6 (27.3)
19. Feeling safe at the institution	82.0 (22.0)	83.7 (21.5)	82.5 (20.9)	83.1 (22.9)	80.2 (23.0)	79.2 (25.1)	76.2 (26.4)
20. Range of available activities satisfactory	63.8 (26.7)	64.4 (28.4)	65.0 (27.4)	68.4 (29.8)	65.2 (28.3)	67.2 (27.8)	70.5 (24.4)
21. Satisfactory meals at institution	73.7 (25.2)	73.1 (24.7)	71.9 (27.2)	75.3 (26.4)	73.9 (26.1)	72.8 (25.4)	71.3 (23.2)
22. Satisfactory level of privacy	76.7 (23.4)	77.1 (23.4)	80.0 (21.7)	77.6 (24.3)	69.1 (26.7)**	74.0 (26.2)	79.6 (21.8)
**Person-Centred Interactions**	66.6 (19.1)	67.8 (17.9)	68.5 (20.7)	70.8 (19.5)	66.6 (18.3)	67.7 (19.5)	69.8 (21.0)
8. Time for discussion/contact with therapist	73.4 (23.1)	73.9 (23.3)	76.5 (24.8)	76.9 (23.3)	79.6 (19.2)	75.2 (22.1)	79.1 (22.0)
9. Therapists/staff understood your situation	74.0 (24.4)	76.1 (21.7)	75.8 (25.0)	77.8 (23.1)	70.1 (27.7)	72.2 (26.0)	74.6 (24.6)
10. Tell important things about condition	75.9 (21.9)	77.8 (22.6)	75.6 (24.4)	79.2 (21.5)	71.8 (24.6)	71.6 (23.8)	76.7 (26.4)
12. Prepared to the time after discharge	62.0 (26.6)	64.4 (28.1)	66.1 (26.5)	69.5 (25.8)**	71.2 (24.4)**	64.4 (27.8)	68.4 (29.7)
13. Treatment adapted to your situation	69.8 (24.9)	70.6 (23.9)	72.3 (25.7)	74.9 (24.9)	69.7 (25.6)	70.2 (25.5)	73.3 (28.7)
14. Influence choice of treatment	61.0 (27.6)	60.2 (27.0)	59.8 (30.4)	63.3 (25.0)	54.2 (31.1)	58.5 (30.0)	65.3 (30.4)
15. Influence choice of medication	61.1 (29.2)	59.5 (29.3)	59.7 (30.0)	61.5 (32.0)	54.7 (33.1)	60.3 (31.7)	59.4 (27.2)
17. Information about mental health/diagnosis	62.6 (27.4)	64.3 (25.1)	65.7 (28.3)	66.2 (28.1)	63.2 (32.6)	62.6 (30.6)	67.7 (31.1)
18. Information about treatment options	59.0 (27.7)	61.6 (27.5)	63.5 (27.5)	68.1 (28.0)***	58.8 (27.8)	65.0 (29.0)*	64.4 (28.7)
**Intermediate outcomes**	64.8 (22.2)	65.8 (22.1)	67.1 (21.7)	67.3 (22.7)	66.0 (22.9)	63.9 (24.0)	65.7 (21.9)
25. Better understand mental health issues	64.3 (26.8)	66.5 (26.4)	69.6 (26.9)	67.6 (27.8)	64.1 (28.0)	64.3 (28.6)	64.8 (28.3)
26. Better cope with mental health issues	61.7 (25.7)	61.5 (26.1)	63.4 (27.6)	66.3 (27.1)	65.8 (27.8)	60.9 (27.4)	66.0 (24.2)
27. Confidence that life will be better	59.8 (27.2)	61.4 (27.1)	59.2 (29.2)	64.6 (28.4)	69.8 (26.9)**	60.0 (30.7)	62.1 (29.6)
28. Overall help been satisfactory	71.7 (23.8)	73.3 (22.6)	76.7 (22.8)	74.0 (23.0)	69.9 (25.1)	70.9 (23.7)	72.2 (26.4)
29. Overall benefit of the treatment	65.9 (25.4)	67.4 (25.7)	66.3 (26.2)	64.3 (25.3)	60.6 (29.7)	60.4 (29.0)*	62.9 (30.6)

^a^Differences between Norwegian-born patients and immigrant groups were investigated using ANOVA

^b^ Tukey post hoc test was used to identify exact differences between groups

* Significant differences between Norwegian-born and the region-of-birth group at the p < 0.05 level

** Significant differences between Norwegian-born and the region-of-birth group at the p < 0.01 level

*** Significant differences between Norwegian-born and the region-of-birth group at the p < 0.001 level

**Table 3:** Coercion, mental health, and mental health change score according to region of birth

*N*(%)	Norway (*N* = 7,325)	Nordic countries without Norway (*N* = 160)	Western Europe/ North America/ Oceania (*N* = 134)	Eastern Europe (*N* = 171)	Africa (*N* = 119)	Asia including Turkey *N* = 212)	South and Central America (*N* = 63)
**Were you admitted against your will?**							
Yes	873 (12.0)	18 (11.3)	18 (13.5)	37 (21.9)*	51 (42.9)*	63 (29.9)*	10 (16.1)
No	6,410 (88.0)	142 (88.8)	115 (86.5)	132 (78.1)	68 (57.1)	148 (70.1)	52 (83.9)
**Have you experienced coercion during this stay (e.g. involuntary admission, forced medication or being restrained)? **							
Yes	803 (11.0)	10 (6.3)	25 (18.7)	28 (16.6)	42 (35.3)*	41 (19.6)*	10 (16.1)
No	6,485 (89.0)	149 (93.7)	109 (81.3)	141 (83.4)	77 (64.7)	168 (80.4)	52 (83.9)
**Is the treatment voluntary or do you feel coerced into receiving it**							
Completely voluntary	4,403 (60.8)	106 (66.7)	67 (51.1)	105 (63.3)	36 (31.0)*	115 (56.1)	41 (66.1)
Rather voluntary	1,282 (17.7)	19 (11.9)	28 (21.4)	38 (22.9)	34 (29.3)*	34 (16.6)	7 (11.3)
Both voluntary and coerced	1,040 (14.4)	27 (17.0)	22 (16.8)	15 (9.0)	24 (20.7)	31 (15.1)	11 (17.7)
Rather coerced	238 (3.3)	6 (3.8)	8 (6.1)	3 (1.8)	8 (6.9)	5 (2.4)	1 (1.6)
Completely coerced	277 (3.8)	1 (0.6)	6 (4.6)	5 (3.0)	14 (12.1)*	20 (9.8)*	2 (3.2)
Mental health							
**Self-perceived mental health the week prior to admission **							
Very poor	3,402 (46.6)	90 (56.3)	61 (45.5)	62 (36.3)	36 (30.5)*	100 (47.4)	23 (37.7)
Rather poor	2,245 (30.8)	50 (31.3)	44 (32.8)	56 (32.7)	24 (20.3)	59 (28.0)	21 (34.4)
Both	1,193 (16.4)	11 (6.9)*	20 (14.9)	35 (20.5)	32 (27.1)*	29 (13.7)	3 (4.9)
Rather good	328 (4.5)	6 (3.8)	7 (5.2)	11 (6.4)	17 (14.4)*	19 (9.0)*	12 (19.7)*
Very good	125 (1.7)	3 (1.9)	2 (1.5)	7 (4.1)	9 (7.6)*	4 (1.9)	2 (3.3)
**Self-perceived mental health at discharge**							
Very poor	735 (10.1)	17 (10.7)	10 (7.5)	12 (7.0)	7 (5.9)	29 (13.7)	4 (6.5)
Rather poor	1,892 (25.9)	33 (20.8)	29 (21.6)	35 (20.5)	14 (11.8)*	36 (17.0)	15 (24.2)
Both	3,029 (41.5)	71 (44.7)	58 (43.3)	63 (36.8)	26 (21.8)*	78 (36.8)	18 (29.0)
Rather good	1,317 (18.1)	29 (18.2)	25 (18.7)	45 (26.3)	47 (39.5)*	52 (24.5)	16 (25.8)
Very good	320 (4.4)	9 (5.7)	12 (9.0)	16 (9.4)*	25 (21.0)*	17 (8.0)	9 (14.5)*
**Change in mental health from one week prior to admission to discharge**							
Very positive	2,044 (28.2)	52 (32.5)	35 (26.5)	36 (21.2)	15 (13.0)*	63 (29.9)	15 (24.6)
Rather positive	3,609 (49.7)	78 (48.8)	69 (52.3)	74 (43.5)	37 (32.2)*	95 (45.0)	29 (47.5)
No change	1,003 (13.8)	20 (12.5)	12 (9.1)	32 (18.8)	31 (27.0)*	28 (13.3)	7 (11.5)
Rather negative	513 (7.1)	7 (4.4)	16 (12.1)	25 (14.7)*	26 (22.6)*	22 (10.4)	8 (13.1)
Very negative	90 (1.2)	3 (1.9)	0 (0.0)	3 (1.8)	6 (5.2)*	3 (1.4)	2 (3.3)

^a^ Differences between Norwegian-born patients and immigrant groups were investigated using Chi square test

* Indicates a significant difference between Norwegian-born and the region-of-birth group at the p < 0.05 level

### Experiences of coercion and perceived mental health

**Fig. 1:**
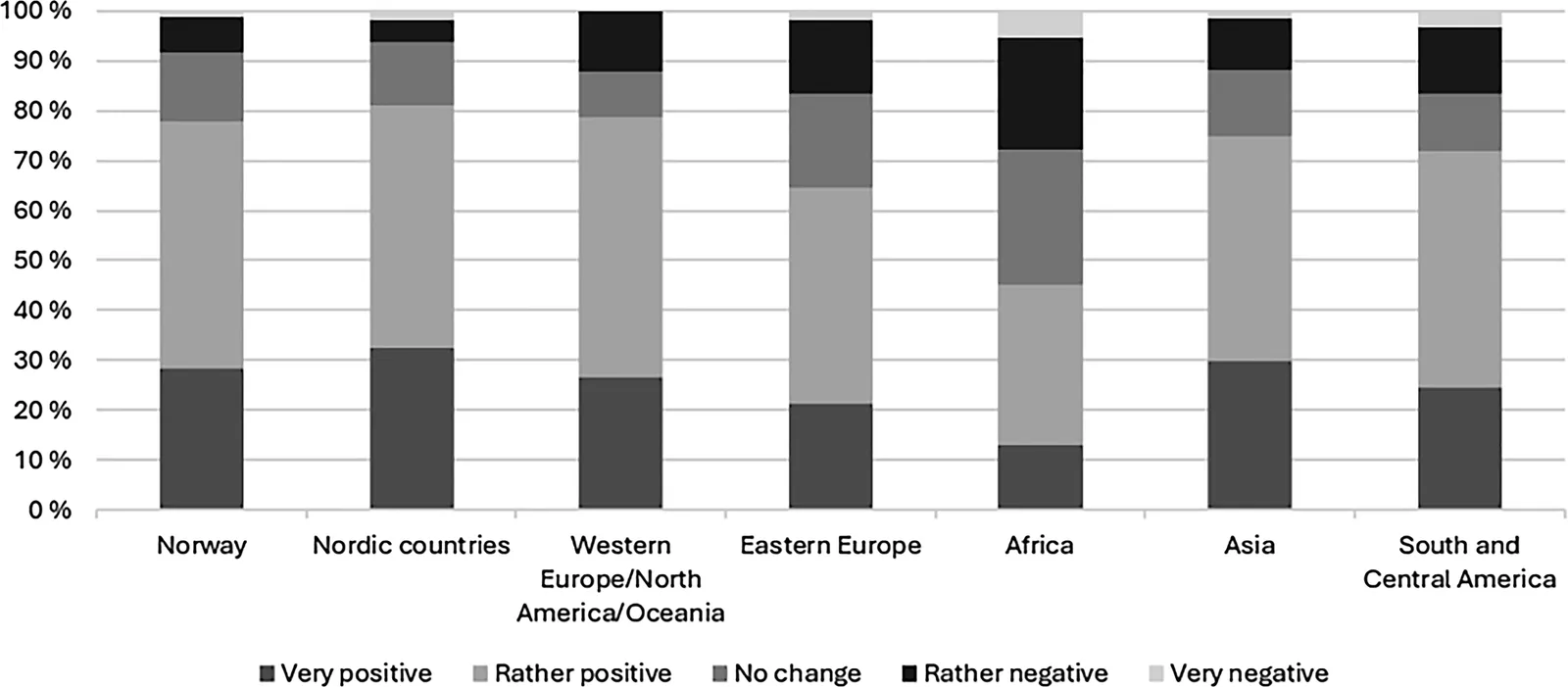
Distribution of mental health change score by region-of-birth groups

Table 3 highlights significant differences between Norwegian-born patients and immigrant groups regarding coercion. Patients from Eastern Europe, Africa, and Asia reported higher rates of involuntary admission. Immigrants from Africa and Asia also reported significantly more experiences of coercion during hospitalization compared to the majority population. In addition, only 31% of patients from Africa considered their treatment completely voluntary, and a significantly higher share of immigrants from Africa (12%) and Asia (10%) stated that treatment was completely coercive compared to the majority population (3.8%). Last, 35.2% of the sample reported poor/very poor mental health at discharge, with patients from Africa reporting significantly higher scores compared to Norwegian-born patients. Patients from Africa and Eastern Europe also reported a significantly less positive trajectory in mental health development, compared to the majority population (Fig. 1).

### Associations between region of birth, patient experiences, coercion, and mental health

Compared to Norwegian-born patients, sex- and age-adjusted multiple linear regression models (Table 4) revealed some significant differences in patient experiences, coercion, and mental health across region of birth. Patients from Africa reported significantly poorer scores for Structure and facilities, more coercion and less positive change in mental health. Furthermore, patients from Eastern Europe had significantly higher scores for Person-centred interactions but less positive change in mental health. Last, patients from Western Europe/North America/Oceania and Asia reported significantly more coercion. Additional adjustments for education, marital status, and the number of prior admissions did not result in notable changes to these findings.

**Table 4:** Multiple linear regression between region of birth, patient experiences, coercion, and mental health change score

Indicators of inpatient experiences with psychiatric care	Structure and facilities	Person-centred interactions	Intermediate Outcomes	Coercion sum score	Mental health change score
*Unstandardized B coefficients and 95% confidence intervals*					
Nordic countries (except Norway)					
Model 1	.159 (-2.56 – 2.88)	.900 (-2.27 – 4.07)	.329 (-3.18 – 3.84)	-.036 (-.17 – .10)	.108 (-.03 – .25)
Model 2	-.119 (-2.82 – 2.58)	.400 (-2.74 – 3.54)	-.379 (-3.85 – 3.09)	-.016 (-.15 – .12)	.112 (-.03 – .25)
Western Europe/North America/Oceania					
Model 1	-.069 (-3.03 – 2.90)	1.559 (-1.89 – 5.01)	1.439 (-2.43 – 5.31)	.167 (.02 – .32)*	-.022 (-.18 – .13)
Model 2	.290 (-2.66 – 3.24)	1.558 (-1.87 – 4.99)	1.052 (-2.79 – 4.89)	.156 (.01 – .30)*	.013 (-.14 – .17)
Eastern Europe					
Model 1	1.804 (-.82 – 4.43)	4.363 (1.38 – 7.35)**	2.765 (-.63 – 6.16)	.055 (-.08 – .19)	-.291 (-.43 – -.15)***
Model 2	1.268 (-1.34 – 3.87)	3.569 (.61 – 6.53)*	1.606 (-1.76 – 4.97)	.085 (-.05 – .22)	-.289 (-.43 – -.15)***
Africa					
Model 1	-3.695 (-6.88 – -.509)*	.289 (-3.29 – 3.87)	1.753 (-2.39 – 5.90)	.683 (.53 – .84)***	-.690 (-.86 – -.52)***
Model 2	-3.512 (-6.67 – -.35)*	.503 (-3.05 – 4.05)	2.166 (-1.93 – 6.26)	.670 (.51 – .83)***	-.692 (-.86 – -.53)***
Asia including Turkey					
Model 1	-1.974 (-4.35 – .406)	1.163 (-1.58 – 3.91)	-.736 (-3.84 – 2.37)	.283 (.16 – .40)***	-.044 (-.17 – .08)
Model 2	-2.233 (-4.60 – .13)	1.015 (-1.71 – 3.74)	-1.014 (-4.09 – 2.06)	.297 (.18 – .42)***	-.042 (-.17 – .08)
South and Central America					
Model 1	.429 (-3.89 – 4.75)	3.285 (-1.71 – 8.28)	.925 (-4.76 – 6.61)	.098 (-.12 – .31)	-.213 (-.44 – .02)
Model 2	.015 (-4.27 – 4.30)	2.919 (-2.03 – 7.87)	.137 (-5.48 – 5.76)	.119 (-.09 – .33)	-.206 (-.43 – .02)

Reference category for region of birth is Norway

Model 1: adjusted for age and sex

Model 2: additionally adjusted for education level, marital status, and number of previous admissions

*p< 0.05

**p< 0.01

***p< 0.001

### Patient comments and main themes

The qualitative analysis was used to contextualize the survey findings and to identify both shared and immigrant-specific aspects of patient experiences. Content analysis identified six shared main themes: [1] Staff attitudes, competence, and availability, [2] The ward environment and everyday life during admission, [3] Treatment content, usefulness, and individual adaptation, [4] Access, continuity, and coordination of care, [5] Perceived changes after admission, and [6] Feeling seen, heard, and involved.

Positive comments about the *Staff attitudes*,* competence*,* and availability* had descriptions of the personnel as kind, empathic, forthcoming and competent, whilst negative comments highlighted experiences with condescending, insensitive, incompetent or busy staff. Positive comments about *The ward environment and everyday life during admission* mentioned a welcoming, calm, socially supportive, safe atmosphere. Negative comments focused on poor facilities and the lack of activities available. Positive comments in *Treatment content*,* usefulness*,* and individual adaptation* described treatment as effective, adapted to own needs, educational, and helpful. Negative comments centred on absent treatment, medication issues, lack of individualized care, and incompetence. Positive experiences about *Access*,* continuity*,* and coordination of care* included early access to care, freedom to move outside, and small stable ward environments. Negative comments addressed long waiting time, premature discharge, lack of information, staff shortages, and poor coordination of appointments. In positive comments about *Perceived changes after admission* patients mentioned getting a better life, having better understanding of the illness, increased stability, and more resilience. On the negative side some patients felt worse off than before admission. Positive comments about *Feeling seen*,* heard*,* and involved* emphasized feeling understood and respected, as well as receiving individualized treatment and training. Negative comments described not being taken seriously, feeling like a burden, or lacking influence over treatment plans and length of stay.

### Immigrant-specific main themes

Three additional themes emerged from 36 comments specifically reflecting immigrant patients’ experiences: [1] Expectations and prior knowledge of psychiatric care, [2] Belonging, trust, and communication with staff and fellow patients, and [3] Finding and understanding information about care, routines, and rights.

### Theme 1 – Expectations and prior knowledge of psychiatric care

The first theme reflected varying expectations and levels of knowledge about psychiatric treatment and the healthcare system. Some patients expressed gratitude for receiving treatment in Norway, whilst others described a discrepancy between their expectations and the reality of care:

I have been admitted to hospital in [country of origin] in the past. The healthcare system in Norway is much better (*Patient from Eastern Europe*)

There was too much downtime here. Patients often stay in their rooms all day alone unless it’s time for medication or meals. This made me sad because I have been in many treatment centers in [country of origin] where there is much more group and individual therapy every single day. (*Western Europe/North America/Oceania*)

Some patients disagreed with the approach to mental health treatment, felt their doctor did not believe them, or wished for greater autonomy. Limited knowledge about the Norwegian healthcare system was also evident:

[I] don’t agree with approaches to personal experiences of illness. More autonomy, less authoritative treatment. More focus on the healthy parts, not just on the sick part. (*Asia*).

Everything was just strange with the whole stay and most of the personnel. (*South and Central America*)

However, being admitted also seemed to increase the patients’ knowledge about the healthcare system as several commented that they had learned a lot, now knew who to contact or the kind of help available to them.

### Theme 2 - Belonging, trust, and communication with staff and fellow patients

The second theme reflected how communication and relationships with staff and fellow patients shaped patients’ sense of belonging and trust. Some patients felt excluded, encountered rude staff, or believed that personnel were not interested in getting to know them. Others struggled to build a trusting relationship with their therapist:

I also had many problems with the night shift nurses on the weekend as they did not get to know us at all and were quite rude to all of us patients. (*Western Europe/North America/Oceania*)

Was left without a therapist because it was a bad match. (*Asia*)

Some patients described language barriers or found communication difficult, stating that what they told staff was not accurately conveyed to the doctor, or that the doctor’s summaries were inaccurate, which made them hesitant to open up:

It was hard for me to open up and it felt like what I told [personnel] didn’t reach the doctor in the same way, so I didn’t feel safe, and it felt pointless. (*Eastern Europe*)

I would have needed a translator during the stay. *(Africa*)

Conversely, some patients felt included and supported by personnel despite language difficulties. Others appreciated being treated as a whole person rather than just a diagnosis.

### Theme 3 - Finding and understanding information about care, routines, and rights

The third theme reflected difficulties finding and understanding information about the inpatient stay, including information about daily routines, practical rules, and rights such as options for appealing decisions.

It took a while before I got the answers I wanted, and the information about the whole stay was somewhat messy. (*Asia*)

Day six, I asked to change two small towels and was told I could do it myself whenever needed. When I asked for a basin on day nine to wash some of my underwear, I was informed that a washing machine was available in the basement. (*Eastern Europe*)

### Summary of results

Results from the quantitative analyses indicated small differences in patient experiences across groups but higher levels of perceived coercion, less positive change in mental health from admission to discharge, or both, among patients from Eastern Europe, Africa, and Asia. Analysis of patient comments revealed that immigrant patients had unique experiences related to expectations and prior knowledge of psychiatric care, belonging and trust in relationships with staff and fellow patients, and finding and understanding information. Many described discrepancies between their expectations and the reality of care, some struggled to build trusting relationships with healthcare personnel, and language barriers and insufficient or unclear information about routines and rights, further complicated their experiences.

## Discussion

This study provides insights into the experiences of immigrant patients in psychiatric inpatient care in Norway. The findings indicate few differences between immigrant groups and the majority population concerning the main patient-reported experience indicators. However, differences were found in single PREM items, coercion and perceived mental health. Patient comments reflected that immigrant patients’ experiences and reporting were related to personal expectations, prior knowledge of psychiatric care, belonging and trust, communication, and access to information.

### Patient-reported experience indicators and main themes from patient comments

Few associations were found between region-of-birth groups and the three main patient-reported experience indicators. These findings were also reinforced by patient comments, which indicated broadly similar experiences across region-of-birth groups. No main themes emerged as unique to the majority population, suggesting that Norwegian-born and immigrant patients shared common concerns and perspectives regarding their care experiences. Similar themes have been identified in previous analysis of patient comments from the psychiatric inpatient setting [32, 33].

These findings are contrary to results from other healthcare services, such as experiences with general practitioners [34–36], child and adolescent mental health services [37] and rehabilitation services for children with disabilities [38]. However, our results align with reviews reporting no consistent results regarding patient experiences and immigrant status in mental health and addiction services [13, 14].

Furthermore, the overall patient-reported experience scores were relatively low compared to results reported in other healthcare services [34]. Previous research shows that the quality of care in mental health has not increased to the same extent as for other areas of medicine [39]. The low ratings of Intermediate outcomes suggest room for improvement, particularly regarding perceived benefit of treatment. However, the complexity of providing inpatient psychiatric care may also contribute to these lower scores.

Despite overall similarities, notable differences between immigrant groups and the majority population were found for coercion. Patients born in Africa, Asia, Eastern Europe, or Western Europe/North America/Oceania reported higher levels of involuntary admissions and/or coercive measures during their inpatient stay. Somewhat surprisingly, the higher levels of coercion among certain immigrant groups did not correspond with lower overall patient-reported experience scores, suggesting a complex relationship between coercion and patient-reported experiences [13, 24]. However, differences in single PREM items, and immigrant-specific patient comment themes provide a more nuanced picture.

### Single PREM items and immigrant-specific themes from patient comments

Patients born in Africa or Asia had lower ratings on single PREM items concerning initial welcome, level of privacy, and overall benefit of the treatment. These item-level differences indicate that some aspects of inpatient care may have been experienced less positively by the same groups that also reported higher levels of coercion. Previous research has shown that involuntary psychiatric admission is associated with clinical and service-related factors, including diagnosis, limited prior contact with psychiatric services, lack of outpatient treatment before hospitalization, and migratory background [22, 23]. The immigrant-specific themes in the present study provide relevant context for interpreting these findings. Limited familiarity with psychiatric care, difficulties finding and understanding information, language and communication challenges, and difficulties establishing belonging, trust, and collaboration may make care appear less transparent, less predictable, or less collaborative. These experiences overlap with barriers previously described for migrant and immigrant patients in mental healthcare [15, 16]. Taken together, these findings suggest that limited familiarity with psychiatric care, communication barriers, and difficulties establishing trust may be particularly relevant for understanding the context of involuntary admission and coercive measures among immigrant patients.

In contrast, immigrant patients from Africa, Asia and Eastern Europe had higher scores on items about receiving information on treatment options, being prepared for the time after discharge, and having confidence in life after discharge. These findings are further supported by patient comments regarding their experiences learning about the healthcare system during their inpatient stay. These findings may reflect differences in knowledge about the healthcare system among immigrant patients [10–12], but may also indicate that healthcare providers are making efforts to increase health literacy.

### Challenges of measuring and comparing patient experiences

Cultural differences may influence patient experiences and survey responses. Prior expectations, as reflected in our findings, shape assessments of experiences [18, 19], while differences in interpretation of questionnaire items and response tendencies may influence patient experience scores across population groups [17, 40]. Additionally, understanding and reporting of mental health may vary across cultural contexts [10, 17, 40]. Family influences and stigma surrounding mental health in certain cultures [10, 12, 41] might lead to underreporting challenges or inflated perceptions of care quality. This trend may be present in the current study, considering the contradictory mental health change scores. These findings underscore the complexity of comparing patient experiences across cultural backgrounds. There is therefore a need for culturally adapted survey methodologies and qualitative approaches to better understand how cultural norms shape healthcare perceptions and patient experiences. Together, these findings suggest that observed differences in patient-reported experiences may reflect not only differences in care, but also differences in how experiences are understood, evaluated, and reported across cultural contexts.

### Overall discussion

The current findings emphasize the importance of examining targeted strategies to reduce involuntary admissions and coercion for immigrant patients born in Africa, Asia and Eastern Europe. Immigrant-specific patient comments highlight the relevance of strengthening patients’ understanding of psychiatric care, building belonging and trust in relationships with staff and fellow patients, and ensuring clear information about care, routines, and rights. Additionally, culturally sensitive care that reduces language barriers, supports trusting relationships, and provides clear practical information may increase patient engagement and involvement in treatment decisions [10, 14–16]. Ensuring accessibility is a critical step towards effective disease management and health outcomes [42], and patient-centred interaction has been found to be a key determinant for patients’ positive assessment of treatment outcomes in psychiatric inpatient care [24].

### Strengths and limitations

This study has several strengths. The use of both survey data and patient comments offers a comprehensive approach [28, 29] to exploring immigrant patients’ experiences with psychiatric care compared to the majority population. Although the qualitative comments provided contextual insight into how patients described their experiences, they should be interpreted as complementary to the survey findings, rather than providing full explanations for the observed group differences.

The large sample size and categorization of immigrant groups by region of birth enhance the study’s ability to provide meaningful insights into this complex issue. However, region of birth remains a crude indicator of migration background and heritage. Immigrants are a highly heterogeneous group, encompassing individuals with diverse migration backgrounds, lengths of residence in Norway, and varying cultural, ethnic, and socioeconomic contexts [6]. A larger sample could allow for a more nuanced understanding of inpatient experiences across more specific immigrant populations.

The composition of immigrant respondents in the present study may reflect selection at two stages. First, barriers to accessing specialist mental healthcare [9–12] may influence which people with an immigrant background access and receive inpatient care and are therefore eligible for the patient experience survey. Second, among eligible patients, the questionnaires were available only in Norwegian and English. This may have affected participation, particularly among patients with lower proficiency in these languages, potentially limiting the generalizability of the findings.

In interpreting the composition of the responding sample, it is also relevant that the immigrant population in Norway differs demographically from the majority population [43], particularly in age structure. Supplementary Table 1 therefore provides contextual information on age, sex, and education in the corresponding general population groups. Although these figures cannot be used to assess the generalizability of the psychiatric inpatient sample directly, the lower proportion of respondents with primary education in the immigrant sample than in the general immigrant population raises the possibility that immigrant patients with lower education were underrepresented in the responding sample. If so, the study findings may underestimate the challenges experienced by more vulnerable immigrant patients.

Determining whether and to what extent such underrepresentation occurred would require information on the number and background characteristics of non-respondents. Such information was unavailable in the present study. Future studies should include information on non-respondents to better assess representativeness and potential selection bias. They should also consider translated questionnaires and multilingual administration, while recognizing the additional logistical and financial resources required.

An additional strength is that the regression analyses adjusted for available background variables, including age, sex, educational level, marital status, and number of previous admissions. The findings remained largely unchanged after adjustment. Nevertheless, unmeasured demographic or clinical differences between Norwegian-born patients and immigrant groups may still have influenced the results. Information on patients’ specific diagnoses was not available, and the hospital- and unit-level information did not allow us to account for differences in areas of specialization. This limits our ability to determine whether the observed group differences partly reflect differences in diagnostic composition or hospital-specific characteristics. At the same time, PIPEQ-CEM is a generic instrument developed for use across psychiatric inpatient populations, supporting the relevance and comparability of the patient-reported experience data across the included groups.

Finally, the anonymous data collection approach means that readmitted patients might have answered the questionnaire more than once. The magnitude of this potential bias could not be estimated. However, each inpatient stay may represent a distinct care episode. Given the cross-sectional design, causal relationships between immigrant background and reported patient experiences cannot be established.

## Conclusion

The findings provide a comprehensive understanding of immigrant inpatients’ experiences in psychiatric care in Norway compared to the majority population. While patient-reported experiences show few differences, disparities in reported coercion were observed among patients born in Africa, Asia, and Eastern Europe. Immigrant-specific themes identified in patient comments suggest that differences in expectations, prior knowledge of psychiatric care, belonging and trust, communication, and access to information may shape care experiences. Together, these findings suggest potential differences in access to healthcare services across patient groups and highlight the importance of culturally appropriate approaches in both care delivery and in measuring and comparing patient-reported experiences to promote equitable mental healthcare. Further research is needed to better understand the unique challenges and experiences of patients from diverse cultural and socioeconomic backgrounds. Addressing systemic barriers and fostering culturally competent practices may contribute to more equitable mental healthcare for all patients.

## Supplementary Information

Below is the link to the electronic supplementary material.

Supplementary Material 1

## Data Availability

The datasets generated and/or analyzed during the current study are not publicly available due to the need to protect personal data but are available from the corresponding author on reasonable request.
