# Supplementary material for: Patient-reported experience measures (PREMs), coercion, and mental health outcomes in psychiatric inpatient care: a national mixed-methods study comparing immigrant and majority populations
Source: BMC Health Serv Res. 2026 Sep 12;26:1317. doi: 10.1186/s12913-026-15511-0 (PMC13615574; doi:10.1186/s12913-026-15511-0)
Supplement: Supplementary file 1 [file 12913_2026_15511_MOESM1_ESM.docx]

Supplementary Table 1. Selected demographic characteristics of the majority and immigrant populations in Norway.

| **Variables** | **Majority population (%)** | **Immigrant population (%)** |
| --- | --- | --- |
| **Sex** |  |  |
| Female | 50.1 | 49.2 |
| Male | 49.9 | 50.8 |
| **Age** |  |  |
| 20-29 | 16.3 | 14.4 |
| 30-39 | 16.2 | 25.3 |
| 40-49 | 13.9 | 27.1 |
| 50-59 | 16.9 | 17.5 |
| 60-66 | 11.1 | 7.7 |
| 67+ | 25.6 | 8.0 |
| **Education** |  |  |
| Primary school | 17.1 | 25.4 |
| Upper secondary school | 42.7 | 31.0 |
| University or college | 40.2 | 43.6 |

^a^ Authors’ calculations are based on data extracted from Statistics Norway’s StatBank, table 09430.

^b^ The majority population comprises Norwegian-born persons with immigrant parents and the remaining population, as defined by Statistics

Norway. This grouping was used to approximate the Norwegian-born comparison group in the study, although the definitions are not

entirely equivalent.

^c^ Immigrants are defined as persons born abroad with two foreign-born parents and four foreign-born grandparents, as defined by Statistics

Norway.

^d^ Age categories were aggregated by the authors based on the categories available in Statistics Norway’s StatBank and do not correspond

exactly to those used for the study sample. The SSB category 16–19 years was excluded because it also includes persons younger than the

eligible study population

^e^ Tertiary vocational education was included in the upper secondary school category, while short and long higher education were combined

as university or college. For immigrants with missing information on educational attainment, Statistics Norway includes estimated

education levels in the figures.
